# Arthroscopic capsular release for the treatment of post-stroke frozen shoulder: A protocol for systematic review

**DOI:** 10.1097/MD.0000000000022025

**Published:** 2020-09-25

**Authors:** Long-ze Zong, Li Ma, Ying-ying Liu

**Affiliations:** a Department of Joint Surgery, Yanan University Affiliated Hospital, Yan’an, China; b Department of Neurology, Yanan University Affiliated Hospital, Yan’an, China; c Third Ward of Neurology Department, Cardiology and Cerebrovascular Specialty Section, Yanan University Affiliated Hospital, Yan’an, China.

**Keywords:** arthroscopic capsular release, efficacy, frozen shoulder, stroke

## Abstract

**Background:**

This study will assess the efficacy and safety of arthroscopic capsular release (ACR) for the treatment of post-stroke frozen shoulder (PSFS).

**Methods:**

We will carry out a systematic study of randomized controlled trials that assess the efficacy and safety of ACR for PSFS. We will search all potential records for any eligible trials from selected electronic databases (MEDLINE, EMBASE, Cochrane Library, Web of Science, Chinese Biomedical Literature Database, WANGFANG, and China National Knowledge Infrastructure) and grey literature sources from inception to the present. Two authors will independently perform study selection, data extraction, and study quality assessment. Any disagreement will be solved by a third author via consultation. Statistical analysis will be carried out by RevMan 5.3 software.

**Results:**

This study will comprehensively summarize current eligible studies to systematically assess the efficacy and safety of ACR for PSFS.

**Conclusion:**

This study will provide evidence to determine whether ACR is an effective management for patients with PSFS.

## 1 Introduction

Stroke is a major health problem worldwide.^[1,2]^ It is also one of the leading causes of serious long-term disability, such as difficulty in swallow, speech problem, urinary or bowel incontinence, depression, anxiety, emotional problems, limbs paralysis, numbness, and pain (including shoulder pain).^[3–8]^ Several studies report that frozen shoulder (FS) may be one of the most substantial reasons of post-stroke FS (PSFS).^[8–12]^ It is reported that about 56.6% stroke patients affect PSFS.^[13]^ In addition, PSFS also can be identified in 77% stroke patients with hemiplegic shoulder pain.^[13,14]^ If such disorder cannot be treated effectively, it greatly affects quality of life in those patients.

Arthroscopic capsular release (ACR) is reported to manage PSFS.^[15–19]^ However, evidence from previous studies has been conflicting, and their results are inconsistent.^[15–19]^ In addition, no existing systematic review examines the efficacy and safety of ACR for the treatment of PSFS. Thus, this is the first systematic review to evaluate the efficacy and safety of ACR for the treatment of PSFS.

## 2 Methods and analysis

### 2.1 Study registration

This study protocol has been registered through INPLASY202070128. We organized it based on the guidelines of the Preferred Reporting Items for Systematic Reviews and Meta-Analysis (PRISRMA) Protocol statement.^[20]^

### 2.2 Inclusion criteria

#### 2.2.1 Types of studies

In this study, we will only consider randomized controlled trials (RCTs) for inclusion, which evaluate the efficacy and safety of ACR for PSFS. Besides RCTs, all other studies will be excluded.

#### 2.2.2 Types of interventions

Patients in the treatment group were treated with ACR alone. Control treatments can be any intervention, such as conventional medication. We will exclude comparators involving ACR.

#### 2.2.3 Types of participants

All participants with a confirmed diagnosis of PSFS will be included. There will be no restrictions regarding the age, sex, country, and other factors.

#### 2.2.4 Types of outcome measurements

The primary outcome is shoulder pain, as measured by any pain scale, such as Numeric Rating Scale.

The secondary outcomes are shoulder function (as evaluated by associated indexes, such as Shoulder Pain and Disability Index), shoulder motion range (as examined by relevant tool, such as Range of Joint Motion Evaluation Chart), shoulder muscle strength (as identified by any tool, such as Cybex Norm isokinetic dynamometer), health-related quality of life (as appraised by any connected questionnaire, such as 36-Item Short Form Survey), and adverse events.

### 2.3 Search strategy

To identify all relevant articles, we will undertake literature search from both electronic databases and grey literature sources to avoid missing potential studies. We will not limit language and publication status. First, we will search the following electronic databases from inception to the present in MEDLINE, EMBASE, Cochrane Library, Web of Science, Chinese Biomedical Literature Database, WANGFANG, and China National Knowledge Infrastructure. We will create search strategy sample of MEDLINE in Table 1. Similar search strategy for other electronic databases will be modified and adapted. Second, we will examine grey literature sources, such as conference proceedings, reference list of included studies, and ongoing trials from websites of clinical trial registry.

**Table 1:**
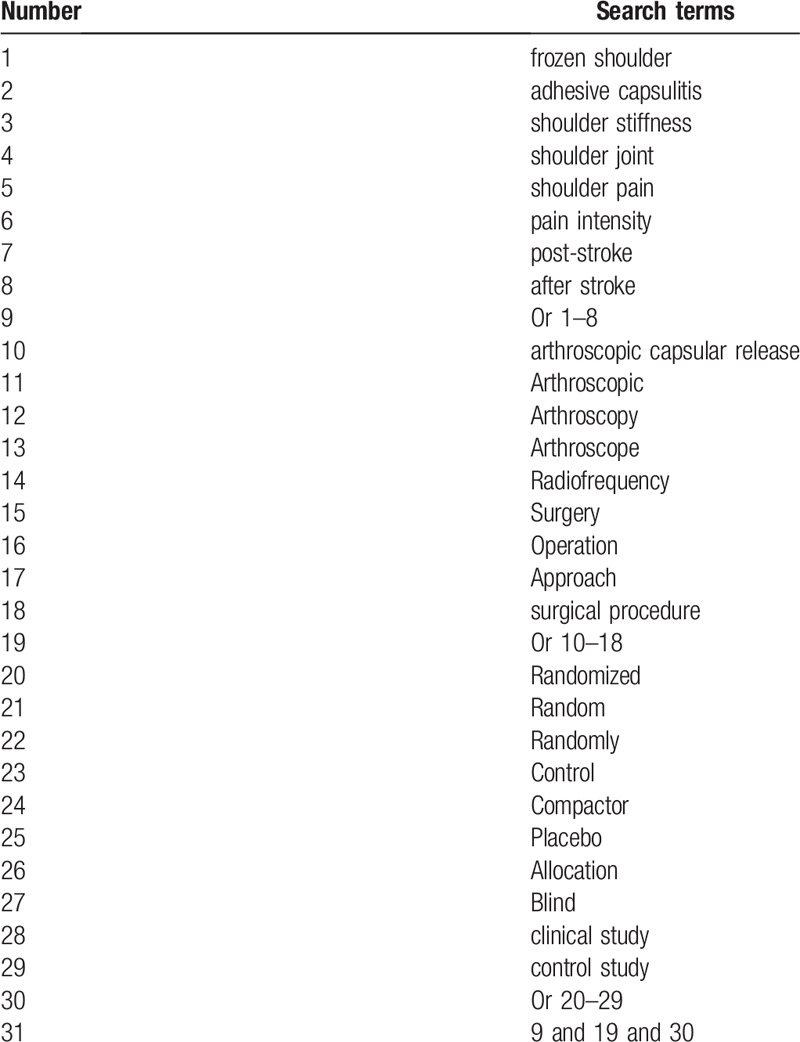
Search strategy of MEDLINE.

### 2.4 Data collection and analysis

#### 2.4.1 Selection of studies

Two authors will independently carry out study selection based on the predefined eligibility criteria. Any division will be solved by a third author through discussion. First, all searched citations will be imported to EndNote X9, and all duplicates will be removed. Second, we will check titles/abstracts of the potential studies, and will eliminate any irrelevant one. Third, we will read full-text of the remaining articles against all inclusion criteria, and all fulfilled studies will be included. We will record all excluded studies with reasons. The results of study selection will be presented in a PRISMA flowchart.

#### 2.4.2 Data extraction and management

Two independent authors will extract data using a pre-designed data extraction form in all eligible trials. Any divergences will be resolved by a third author through consultation. The extracted data comprise of title, first author, publication time, patient characteristics, trial design, trial setting, sample size, details of interventions and controls, outcome indicators, results, conclusion, follow-up information, conflict of interest, and other essential data.

#### 2.4.3 Missing data dealing with

We will contact original trial authors to obtain any unclear or missing data if it occurs. Otherwise, we will analyze available data and will discuss its potential affects to this study.

### 2.5 Study quality assessment

Two authors will independently assess study quality of each eligible trial using Cochrane Risk of Bias Tool. We will appraise each study through 7 aspects, and each one will be valued as low, unclear, or high risk of bias. Any different views will be figured out with the help of a third author through discussion.

### 2.6 Statistical analysis

We will perform statistical analysis using RevMan 5.3 software. All continuous outcome indicators will be expressed using weighted mean difference (MD) or standard MD with 95% confidence intervals (95% CIs), and all dichotomous outcome indicators will be estimated using risk ratio with 95% CIs. We will check heterogeneity across included trials using *I*^*2*^ statistic. *I*^*2*^ ≤50% indicates acceptable heterogeneity, and we will use a fixed-effects model. *I*^*2*^ > 50% suggests remarkable heterogeneity, and we will employ a random-effects model. Whenever necessary under acceptable heterogeneity, we will carry out a meta-analysis based on the sufficient similarity in study information, patient characteristics, details of intervention and control, and study quality. Otherwise, if we identify considerable heterogeneity, we will conduct a subgroup analysis to explore its sources. If a meta-analysis is deemed not to be undertaken, we will report study results using a narrative summary.

### 2.7 Additional analysis

#### 2.7.1 Subgroup analysis

We will undertake a subgroup analysis according to the different study information, participant patient characteristics, variations of intervention and control, and study quality.

#### 2.7.2 Sensitivity analysis

We will conduct a sensitivity analysis to test the robustness of the merged outcomes by excluding trials with low quality.

#### 2.7.3 Reporting bias

We will examine reporting bias using funnel plot and Egger regression test when over 10 RCTs are eligible on the same outcome indicator.^[21,22]^

### 2.8 Grading the quality of evidence

The quality of evidence for all outcome indicators will be appraised using the Grading of Recommendations Assessment, Development, and Evaluation.^[23]^ Each outcome indicator will be graded into 4 levels: high, moderate, low, and very low quality.

### 2.9 Ethics and dissemination

This study will not need ethical approval, because it will not collect individual patient data. We expect to publish this study on a peer-reviewed journal or a relevant conference or meeting.

## 3 Discussion

PSFS is one of the most common complications in stroke survivors, which greatly affect quality of life for them. Therefore, effective managements are needed to treat PSFS. Numerous studies reported that ACR has been used for treating PSFS effectively. However, there is no systematic review specifically relevant to ACR for PSFS, which may restrict its clinical application. Thus, this study will first investigate the efficacy and safety of ACR for PSFS. Its results may provide robust evidence for both clinical practice and patients

## Author contributions

**Conceptualization:** Long-ze Zong, Ying-ying Liu.

**Data curation:** Long-ze Zong, Li Ma, Ying-ying Liu.

**Formal analysis:** Long-ze Zong, Li Ma, Ying-ying Liu.

**Investigation:** Ying-ying Liu.

**Methodology:** Li Ma.

**Project administration:** Ying-ying Liu.

**Resources:** Long-ze Zong, Li Ma.

**Software:** Long-ze Zong, Li Ma.

**Supervision:** Ying-ying Liu.

**Validation:** Long-ze Zong, Li Ma, Ying-ying Liu.

**Visualization:** Long-ze Zong, Li Ma, Ying-ying Liu.

**Writing – original draft:** Long-ze Zong, Li Ma, Ying-ying Liu.

**Writing – review & editing:** Long-ze Zong, Ying-ying Liu.
